# Efficacy and safety of infliximab induction therapy in Crohn's Disease in Central Europe - a Hungarian nationwide observational study

**DOI:** 10.1186/1471-230X-9-66

**Published:** 2009-09-10

**Authors:** Pál Miheller, Péter L Lakatos, Gábor Horváth, Tamás Molnár, Tamás Szamosi, Zsófia Czeglédi, Ágnes Salamon, József Czimmer, György Rumi, Károly Palatka, Mária Papp, Zsolt Jakab, Andrea Szabó, András Gelley, László Lakatos, Zsolt Barta, Csaba Balázs, István Rácz, Margit Zeher, Zoltán Döbrönte, István Altorjay, Béla Hunyady, László Simon, János Papp, János Banai, Ferenc Nagy, János Lonovics, László Újszászy, Györgyi Műzes, László Herszényi, Zsolt Tulassay

**Affiliations:** 1 2nd Department of Medicine, Semmelweis University, Budapest, Hungary; 2 1st Department of Medicine, Semmelweis University, Budapest, Hungary; 3 1st Department of Medicine, Semmelweis Hospital, Miskolc, Hungary; 4 1st Department of Medicine, Faculty of Medicine, University of Szeged, Szeged, Hungary; 5 Department of Internal Medicine, National Medical Center, Budapest, Hungary; 6 2nd Department of Internal Medicine, Balassa János Hospital, Szekszárd, Hungary; 7 1st Department of Medicine, University of Pécs, Faculty of Medicine, Pécs, Hungary; 8 2nd Department of Medicine, University of Debrecen, Faculty of Medicine, Debrecen, Hungary; 9 2nd Department of Internal Medicine, Markusovszky Hospital, Szombathely, Hungary; 10 1st Department of Medicine, Petz Aladár Hospital, Győr, Hungary; 11 Department of Internal Medicine, Polyclinic of the Hospitaller Brothers of St John of God, Budapest, Hungary; 12 1st Department of Medicine Csolnoky Ferenc Hospital, Veszprém, Hungary; 13 3rd Department of Medicine, University of Debrecen, Faculty of Medicine, Debrecen, Hungary

## Abstract

**Background:**

Infliximab (IFX) has proven to be an effective addition to the therapeutic arsenal for refractory, fistulizing, and steroid dependent Crohn's disease (CD), with efficacy in the induction and maintenance of clinical remission of CD. Our objective in this study is to report the nationwide, multicenter experience with IFX induction therapy for CD in Hungary.

**Methods:**

During a 6-year-period, beginning in 2000, a total of 363 CD patients were treated with IFX as induction therapy (5 mg/kg IFX infusions given at week 0, 2 and 6) at eleven centers in Hungary in this observational study. Data analysis included patient demographics, important disease parameters and the outcome of IFX induction therapy.

**Results:**

Three hundred and sixty three patients (183 women and 180 men) were treated with IFX since 2000. Mean age was 33.5 ± 11.2 years and the mean duration of disease was 6.7 ± 6.1 years. The population included 114 patients (31.4%) with therapy-refractory CD, 195 patients (53.7%) with fistulas, 16 patients (4.4%) with both therapy-refractory CD and fistulas, and 26 patients (7.2%) with steroid dependent CD. Overall response rate was 86.2% (313/363). A higher response rate was observed in patients with shorter disease duration (p = 0.05, OR:0.54, 95%CI:0.29-0.99) and concomitant immunosuppressant therapy (p = 0.05, OR: 2.03, 95%CI:0.165-0.596). Concomitant steroid treatment did not enhance the efficacy of IFX induction therapy. Adverse events included 34 allergic reactions (9.4%), 17 delayed type hypersensitivity (4.7%), 16 infections (4.4%), and 3 malignancies (0.8%).

**Conclusion:**

IFX was safe and effective treatment in this cohort of Hungarian CD patients. Based on our experience co-administration of immunosuppressant therapy is suggested in patients receiving IFX induction therapy. However, concomitant steroid treatment did not enhanced the efficacy of IFX induction therapy.

## Background

Infliximab (IFX) is a chimeric monoclonal antibody that binds soluble and membrane bound tumor necrosis factor-alpha (TNF-α). Previous studies have demonstrated its efficacy in refractory, fistulizing, and steroid dependent Crohn's disease (CD) [1,2]. Furthermore, other studies have shown its efficacy as maintenance therapy for CD [3], moreover as induction and maintenance therapy for ulcerative colitis [4].

In Hungary, IFX has been used in clinical studies since 2000, and has served as an important component of the therapeutic arsenal for the treatment of CD since 2003. Financial considerations limit the use of IFX treatment for CD to eleven Hungarian Gastroenterological Centers which are licensed for its administration. Initially, IFX was available only for induction therapy for patients suffering from fistulizing, therapy resistant, or steroid dependent CD. However, as clinical evidence demonstrates the efficacy of IFX for maintenance therapy [3], currently IFX is available for maintenance therapy as well. The objective of this observational study is to report the nationwide, multicenter experience with IFX induction therapy for CD in Hungary.

## Methods

This observational study was initiated in 2004, therefore a portion of the patient data (2000 through 2004) are retrospective. Patient data through six years from eleven Hungarian Gastroenterological Centers are included in this analysis. Microsoft^® ^Excel databases were used to compile and process patient data including demographic characteristics, localization and behaviour of the disease, concomitant medication, indication for biological therapy and outcome of IFX induction therapy were registered retrospectively. The study protocol was reviewed and approved by the Semmelweis University Regional and Institutional Committee of Science and Research Ethics.

Patients with Crohn's Disease eligible for this observational study had 1) single or multiple discharging abdominal and/or perianal fistulas of at least 3-6 months duration despite conventional immunosuppressant and antibiotic therapy; 2) therapy refractory/steroid dependent luminal disease. Minority of patients had active luminal and fistulizing disease as well. Patients who are either unable to reduce corticosteroids below the equivalent of prednisolone 10 mg/day within three months of starting corticosteroids without recurrent active disease, or who have a relapse within three months of stopping corticosteroids were defined as corticosteroid dependent patient. Corticosteroid refractory disease was defined as active disease despite prednisolone up to 0.75-1 mg/kg/day over a period of four weeks defined as steroid refractory. Patients who not tolerate AZA in a dose at least 1.5 mg/kg body weight were considered as AZA intolerant. Failure of immunosupressants (AZA or else) therapy or AZA intolerance was an indication for IFX therapy also.

As induction therapy five mg/kg body weight of infliximab was administered in a 2 hour infusion at weeks 0, 2 and 6. In fistulizing disease response was defined as a decrease of 50% or more in the number of discharging fistulas compared to baseline and remission was defined as absence of any discharging fistulas measured at week 12. In patients with therapy-refractory or steroid dependent luminal disease the response was defined as a ≥70 points decrease in CDAI and a CDAI value below 150 was considered as clinical remission at week 12. The Crohn's Disease Activity Index (CDAI) was calculated only in cases with steroid dependent or therapy-refractory luminal disease.

Infliximab 5 mg/kg body weight was given as maintenance therapy after week 6. Maintenance was given every 8 weeks. The initiation of the maintenance therapy was based on the assessment of response following the induction regimen. However, financial restrictions prevented a significant proportion of patients from receiving maintenance therapy. Conventional induction therapy (1 mg/kg body weight steroid therapy) was started in case of early disease relapse in patients with luminal CD. In case of late relapse (defined as relapse after at least 6 months of remission) IFX re-induction regimen was administered.

### Statistcal methods

Variables were tested for normality using Shapiro Wilk's W test. T-test with separate variance estimates, ANOVA with *post hoc *Scheffe test, χ^2^-test, and χ^2^-test with Yates correction were used to evaluate differences within subgroups of IBD patients. The results are presented as means ± SD. Association between response, remission and clinical variables (with variables with a p < 0.2 in univariate analysis) was also tested by using logistic regression analysis. A *p *value of < 0.05 was considered as significant. For the statistical analysis, SPSS15.0 (SPSS Inc, Chicago, IL) was used.

## Results

During a 6-year-period 363 CD patients were treated with IFX. The cohort comprised 183 females and 180 males; the mean age was 33.5 ± 11.2 years and the mean duration of disease was 6.7 ± 6.1 years at the time of initiation of induction therapy.

One hundred and ninety five (53.7%) fistulizing, 114 (31.4%) therapy-refractory, and 26 (7.2%) steroid dependent CD patients were treated. All patients were naïve to TNFα-inhibitor therapy. Five (0.1%) patients with metastatic CD and 7 (0.2%) patients with extra-intestinal manifestations were also treated, but these patients were not available for assessment of response. The details regarding the specific indications for IFX therapy are summarized in Table 1.

**Table 1:** Indications and response rates of infliximab induction therapy

Indication of IFX therapy	Number and rate of disease type	Response rate after induction
*Therapy-refractory CD*	114 (31.4%)	83.3%
By localization		

Ileum	10 (8.77%)	
Colon	29 (25.4%)	79.3%
Ileo-colonic	46 (40.4%)	87.0%
Ileo-colonic and small bowel	28 (24.6%)	78.6%
Oesophagus	1 (0.9%)	1/1

*Therapy-refractory and fistulizing*	16 (4.4%)	93.8%

*Fistulizing*	195 (53.7%)	85.6%
By localization		

Perianal	148 (75.9%)	91.2%
Enterocutaneous	24 (12.3%)	87.5%
Enterovaginal	11 (5.6%)	9/11
Other	12 (6.2%)	7/12
Mixed	7 (3.6%)	5/7

*Steroid dependent*	26 (7.2%)	92.3%
By localization		

Ileum	3 (11.5%)	3/3
Colon	4 (15.4%)	4/4
Ileo-colic	19 (73.1%)	17/19

*Metastatic*	5 (0.1%)	Na.

*Other*	7 (0.2%)	Na.

Na = not applicable

During the observation period 1,532 IFX infusions were administered. Complete induction regimen was performed in 299 patients (82.3%). Overall response rate was 86.2% thus 313 patients out of the 363 responded to the induction therapy. Details regarding the response rates are shown in Table 1. The overall remission rate was 46.0% (167 out of 363 patients) after IFX induction therapy. Detailed data on remission rates are shown in Table 2.

**Table 2:** Remission rate after infliximab induction therapy

Indication of IFX therapy	Number and rate of remission	
Overall	167 (46%)	

*Therapy-refractory CD* by localization	45 (39.47%)	

Ileum	3/10 (30%)	
Colon	7/29 (24.13%)	
Ileo-colonic	29/46 (63.04%)	
Ileo-colonic and other small bowel	5/28 (17.85%)	
Oesophagus	1/1	

*Therapy-refractory and fistulizing*	6 (37.5%)	

Fistulizing	95 (48.71%)	

Perianal	72/148 (48.64%)	
Enterocutaneous	7/24 (29.16%)	
Enterovaginal	3/11	
Other	4/12	
Mixed	6/7	

Steroid dependent	15 (57.69%)	

Ileum	3/3	
Colon	3/4	
Ileo-colic	9/19 (47.36%)	

Metastatic	5	Na.

Other	1	Na.

Na = not applicable

Among 304 (83.7%) patients treated simultaneously with immunosuppressants, 268 responded (88.2%), and 36 failed to respond (11.8%). Fifty nine (16.2%) patients did not receive any concomitant immunosuppressants and the response rate was lower in these patients respect to those on concomitant immunosuppressive therapy (73% vs 88.2%, p < 0.05). Among patients with concomitant immunosuppressants, azathioprine (AZA) was used most frequently (78.9%). Other immunosuppressant therapies included methotrexate (5.9%), and only one patient (0.3%) was treated with cyclosporine and another with mycophenolate mofetil (0.3%). Response rate to IFX induction therapy was higher in patients receiving concomitant AZA or methotrexate (88.3% vs. 76.6%, p = 0.014).

During induction therapy co-administration of pre-infusion corticosteroid therapy was applied in 165 patients. Response rates were similar for patients with or without concomitant steroid treatment (88.5% vs. 85.8%, respectively, p = NS).

Patients who achieved remission or response after induction therapy were significantly younger than patients classified as non-responders (26.8 ± 10.4 years vs. 33.2 ± 11.5 years of age, p < 0.01), furthermore the duration of CD was shorter (5.9 ± 5.5 years vs. 7.2 ± 4.3 years, p < 0.001).

Abdominal surgery prior to IFX therapy was performed in 25.1% of CD patients. This subset of patients was older in age (35 ± 10.9 years of age, p = 0.01), and the mean duration of disease was longer (8.5 ± 6.3 years, p < 0.0001) compared to the entire group of patients. Among patients with previous surgical intervention 75% responded to IFX induction therapy.

Out of 167 patients in remission 136 (81.4%) received simultaneous immunosuppressive regimen. The majority of patients in remission receiving concomitant immunosuppressants were treated with AZA (132 patients, 97.0%), 4 patients (2.9%) received methotrexate, and mesalazine or sulphasalazine was used in 128 (93.4%) and 8 (5.9%) of them respectively.

A logistic regression analysis was performed to test the association between clinical variables, concomitant medical therapy and remission and response to IFX induction treatment (Table 3.). Duration (*p *= 0.05, OR: 0.54, 95%CI: 0.29-0.99) and concomitant steroid therapy (*p *= 0.027, OR: 0.54, 95%CI: 0.31-0.93) were associated with remission at week 12 in the same logistic regression analysis.

**Table 3:** Logistic regression: Predictive factors for response to IFX induction therapy at week 12 in Crohn's disease

Factor	Coefficient	*P *value	OR	95% CI
Gender	0,070	0.828	-	-

Longer disease duration (≤ 10 years vs. > 10 years)	-1.161	< 0.001	0.349	0.165-0.596

Disease behavior	-0,312	0.349	-	-

Concomitant AZA/methotrexate use	0.710	0.05	2.03	1.001-4.168

Steroid use	-0,438	0.184	-	-

The coefficient is equivalent to the natural log of the OR; *p *value: level of significance;

OR: odds ratio; 95% CI: 95% confidence interval.

Adverse events during induction treatment were observed in 78 patients (21.5%, 3.86/100 patient-years). Allergic reactions were detected in 34 patients (43.6% of all adverse events, 1.56/100 patient-years) including 4 events progressing to anaphylactic shock - IFX therapy was immediately discontinued in these cases. Patients suffering from mild to moderate allergic reactions were able to finish the IFX therapy by pre-administering parenteral corticosteroids. Despite previous severe allergic reactions, in 2 patients successful desensitization was performed with stepwise diluted IFX and they were subsequently re-challenged with continuation of their IFX induction therapy.

Delayed type hypersensitivity reactions (DTHR), characterized by muscle pain, fever, joint pain and skin rashes were observed in 17 patients (0.78/100 patient-years). In five of these cases the DTHR occurred after the 2^nd ^infusion of IFX induction regimen, and only one patient was able continue IFX therapy after resolution of the DTHR.

Infections were observed in 16 patients (4.4%, 0.73/100 patient-years) including serious infections in 5 patients (0.01%, 0.23/100 patient-years) such as two cases of tuberculosis, two intra-abdominal abscesses, and one meningeal Listeriosis. The majority of infections (11/16, 68.8%) occurred in patients treated with concomitant immunosuppressants. No fatal infectious complications were observed.

In four cases symptoms of gut stenosis developed after IFX therapy (0.55%, 0.011/100 patient-years), two cases required surgery.

Three cases of malignant solid tumors were observed (0.82%, 0.137/100 patient-years). The first case was diagnosed on the 2^nd ^week of therapy, and was considered unrelated to IFX treatment. In this case, an abdominal abscess was the initial diagnosis because of high fever and diffuse, sharp abdominal pain. However, the diagnosis of colon malignancy was confirmed based on histological evaluation of a tissue sample. In the second case the malignancy was diagnosed in a patient 5 month after the start of induction therapy. This patient had severe therapy-resistant and fistulizing CD with a 15-year disease duration, and was diagnosed with rectal carcinoma resulting in multiple mesenterial adenocarcinoma metastases. The patient died a few months after the diagnosis. The third malignant solid tumor observed in this cohort was a lung cancer. The patient was diagnosed 4 months after the start of induction therapy, although chest X-rays obtained prior to the start of IFX induction therapy did not show any evidence of solid tumor. Initially, the patient was observed for fever, a bad cough and swelling of lower extremities. Computer tomography of the chest revealed the tumor, which was diagnosed histologically as a squamous cell carcinoma. The overall mortality rate was 0.82% (0.137/100 patients-years).

## Discussion

The results of this observational study of CD patients treated at eleven Gastroenterology Centers in Hungary confirm the efficacy of IFX induction therapy reported in previous investigations [5,6]. Data collection was partially retrospective which was one of the limitations of the study. Although, all centers had to have collect some data regarding their patients because of financial requirement. These kinds of data were collected in the retrospective and prospective phase also.

Overall response and remission rates observed in this study were 86% and 46%, respectively. Notably, the response rate was higher among younger patients, and in patients with a shorter duration of CD. Among patients with perianal and enterovaginal fistulas a high response rate were observed after IFX therapy, which is in concordance with previously reported data [7,8].

Higher response and remission rates after induction IFX therapy were observed in patients receiving concomitant therapy with immunosuppressants. In Hungary AZA is the most commonly used immunosuppressant. Given that there are little data on co-administration of other immunosuppressants would improve the therapeutic benefits of IFX in CD [9], we conclude that AZA would be an appropriate first choice [10]. In the present study early response was higher in patients with concomitant AZA, while remission rates at week 12 were higher in patients receiving steroids. The combined effect of the two drugs was not investigated. In addition shorter disease duration was associated with a higher response and remission rate in a logistic regression analysis. Recent results in SONIC study confirms our experiences, but only in AZA naïve patients [11]. In contrast, disease location or behavior were not independent predictors for early response or remission.

Based on our experience IFX therapy was effective therapy for patients with metastatic CD or intractable skin manifestations. In some cases we achieved significant improvement of the symptoms, in accordance with results observed by others [12].

Based on the further data collection a single induction IFX therapy may maintain the remission in a relatively high proportion of patients with luminal disease [13].

The most common adverse events were allergic reactions. In our Gastroenterology Centers, steroid pre-medication was not routinely administered at the initiation of induction therapy. However, in case of allergic reactions re-treatment was attempted applying parenteral corticosteroid administration and slower infusion rate of IFX according to recommendations of Sandborn et al. [14]. Farrel et al. [15] found lower anti-infliximab antibody (ATI) formation after intravenous hydrocortisone pre-medication. Based on these findings and our experience, regular steroid pre-medication remains questionable when regular immunosuppressant therapy is administered in parallel.

Two patients despite severe allergic reaction were further treated with IFX based on the instructions of Duburgue et al [16]. Applying this method we were able to continue maintenance therapy in one patient, however in the second patient, the second dilution series lead to an allergic reaction.

The diagnosis of delayed type hypersensitivity reaction must be considered in case of fever, muscle or joint pain and rash appear several weeks after IFX therapy [17]. Increasing the dose of immunosuppressant and introducing corticosteroid pre-medication was effective and safe in one of our patients experiencing a delayed hypersensitivity reaction [18].

Five patients experienced serious infection and all were treated with broad spectrum antibiotics. One of these patients with meningitis caused by *Listeria monocytogenes *had to be further treated in intensive care unit.

In two patients tuberculosis was diagnosed (0.55%, 0.09/100-patient years). Based on our experience the observed tuberculosis incidence is higher among patients treated with IFX and other immuosupressive co-medication than the reported average 0.02% tuberculosis incidence in Hungary in 2005 [19]. Tuberculin skin tests were applied in all patients before initiating IFX therapy. Results of the tests were hardly interpretable because the immunization of a newborn is obligatory in Hungary. It was important to take into consideration the social circumstances and family history of the patients in this situation.

General incidence of infections was also reported to be higher in IFX treated patients [20], but IFX treatment itself did not predispose to infections [21]. We conclude that performing a tuberculin test and chest x-ray should be mandatory in all situations before anti-TNF therapy. Furthermore, regular x-ray examination is suggested for IFX-treated patients in geographic areas with high prevalence of tuberculosis. Overall mortality rate was similar to that calculated from the database of the TREAT registry [20].

## Conclusion

IFX was safe and effective as induction therapy in this cohort of Hungarian CD patients. Based on our experience in 11 Gastroenterology Centers in Hungary, co-administration of immunosuppressant therapy and shorter disease duration was associated with significantly improved response/remission rates suggested in patients receiving IFX induction therapy.

## Competing interests

The authors declare that they have no competing interests.

## Authors' contributions

This study represents a nationwide experience concerning infliximab induction therapy in Crohn's disease. All of the authors work tertiary reference centers which are able to administer infliximab in Hungary. PM, PLL, GM, TM, TS, ZC, ÁS, JC, GR, KP, MP, ZJ, AS, AG, LL, ZB, CB, IR, MZ, ZD, IA, BH, LS, JP, FN, JL, LÚ, GM, LH, ZT collected data regarding their infliximab treated patients and participated in data processing as well and manuscript preparation. PM, TM, GH, PK, ZT and PLL were actively participating in the design of the trial and database construction. Statistical analysis was performed by PLL and PM. All authors've read and approved the final manuscript.

## Pre-publication history

The pre-publication history for this paper can be accessed here:

http://www.biomedcentral.com/1471-230X/9/66/prepub
